# Prediction of the Molecular Mechanisms Underlying Erlong Zuoci Treatment of Age-Related Hearing Loss via Network Pharmacology-Based Analyses Combined with Experimental Validation

**DOI:** 10.3389/fphar.2021.719267

**Published:** 2021-11-23

**Authors:** Qing Liu, Ning Li, Yifang Yang, Xirui Yan, Yang Dong, Yinting Peng, Jianrong Shi

**Affiliations:** ^1^ School of Basic Medical Sciences, Shanghai University of Traditional Chinese Medicine, Shanghai, China; ^2^ Experimental Teaching Center, Shanghai University of Traditional Chinese Medicine, Shanghai, China

**Keywords:** erlong zuoci, age-related hearing loss, network pharmacology, experimental validation, cellular senescence, inflammatory response, synaptic connections

## Abstract

**Background:** The traditional Chinese medicine formula ErLong ZuoCi (ELZC) has been extensively used to treat age-related hearing loss (ARHL) in clinical practice in China for centuries. However, the underlying molecular mechanisms are still poorly understood.

**Objective:** Combine network pharmacology with experimental validation to explore the potential molecular mechanisms underlying ELZC with a systematic viewpoint.

**Methods:** The chemical components of ELZC were collected from the Traditional Chinese Medicine System Pharmacology database, and their possible target proteins were predicted using the SwissTargetPrediction database. The putative ARHL-related target proteins were identified from the database: GeneCards and OMIM. We constructed the drug-target network as well as drug-disease specific protein-protein interaction networks and performed clustering and topological property analyses. Functional annotation and signaling pathways were performed by gene ontology and Kyoto Encyclopedia of Genes and Genomes enrichment analysis. Finally, *in vitro* experiments were also performed to validate ELZC’s key target proteins and treatment effects on ARHL.

**Results:** In total, 63 chemical compounds from ELZC and 365 putative ARHL-related targets were identified, and 1860 ARHL-related targets were collected from the OMIM and GeneCards. A total of 145 shared targets of ELZC and ARHL were acquired by Venn diagram analysis. Functional enrichment analysis suggested that ELZC might exert its pharmacological effects in multiple biological processes, such as cell proliferation, apoptosis, inflammatory response, and synaptic connections, and the potential targets might be associated with AKT, ERK, and STAT3, as well as other proteins. *In vitro* experiments revealed that ELZC pretreatment could decrease senescence-associated *β*-galactosidase activity in hydrogen peroxide-induced auditory hair cells, eliminate DNA damage, and reduce cellular senescence protein p21 and p53. Finally, Western blot analysis confirmed that ELZC could upregulate the predicted target ERK phosphorylation.

**Conclusion:** We provide an integrative network pharmacology approach, in combination with *in vitro* experiments to explore the underlying molecular mechanisms governing ELZC treatment of ARHL. The protective effects of ELZC against ARHL were predicted to be associated with cellular senescence, inflammatory response, and synaptic connections which might be linked to various pathways such as JNK/STAT3 and ERK cascade signaling pathways. As a prosperous possibility, our experimental data suggest phosphorylation ERK is essential for ELZC to prevent degeneration of cochlear.

## Introduction

As reported by WHO, hearing loss currently affects more than 1.5 billion people worldwide (Chadha et al., 2021). Age-related hearing loss (ARHL), also called presbycusis, is characterized by gradual hearing loss, particularly for higher frequencies, in the aging population (Gates and Mills, 2005; Yamasoba et al., 2013). The prevalence of ARHL increased with age, particularly after the mid-50s. Approximately 40% of individuals aged 65 or older have impaired hearing (Patel and McKinnon, 2018), which can substantially contribute to depression, social isolation, and a high risk of dementia (Loughrey et al., 2018; Deal et al., 2021). The pathophysiological mechanisms involved in ARHL include oxidative stress, excitotoxicity, inflammation, and ischemia, resulting in age-related changes in specific locations within the cochlea (auditory hair cells, stria vascularis, spiral ganglion neurons, and supporting cells) (Pool and Meagher, 1990; Mazelova et al., 2003). Most cases of ARHL exhibit a mixture of several pathological changes. The molecular mechanisms underlying pathophysiological processes that were reported in recent years include pathways related to antioxidant, DNA damage and DNA damage responses, mitochondrial dysfunction, senescence, and cell death (apoptosis and autophagy). However, the precise molecular mechanism underlying the ARHL remains unclear. Currently, no effective medication is available to prevent or treat ARHL (Wang and Puel, 2020). The complexity of the mechanisms underlying the various forms of hair cells, neural, and/or striatal degenerations escape from simple target therapy, thus a more synthetic treatment is needed. For example, the potential role of oxidative stress in ARHL has aroused researchers’ significant interest in clinical trials investigating dietary antioxidants which target oxidative stress pathways, whereas the outcomes are controversial (Sha et al., 2012; Tavanai and Mohammadkhani, 2017). The antioxidant seems to be a necessary but insufficient preventive option for the ARHL. Targeting several pathways might be better than targeting the only oxidative stress pathway (Tavanai and Mohammadkhani, 2017).

In traditional Chinese medicine, the aging process (including presbycusis) results from a deficiency of kidney essence, the fundamental substance supporting human life. Therefore, kidney-nourishing therapy has been widely applied to different age-related diseases in Chinese clinical settings. Erlong Zuoci (ELZC) decoction, which consists of 7 botanical drugs and *Magnetitum*, is a classic kidney-tonifying prescription that is now available for treating hearing loss in Chinese communities. Despite the lack of large-scale clinical studies, some clinical observation studies have shown that ELZC may restore hearing loss in the elderly (Xie and Zheng, 2017; Hu et al., 2019; Zou, 2020). A very recent clinical trial of ARHL patients treated with ELZC showed a higher effective rate in the treatment group (ELZC + Standard Sound Therapy) than the control group (Standard Sound Therapy) (86.67 vs. 60%). The frequency of complete hearing recovery at day 90 was 36.67% in the treatment group, and 16.67% in the control groups, respectively. No serious adverse events were reported (Zou, 2020). There is evidence that ELZC could protect cochlear hair cells from gentamicin-induced ototoxicity (Dong et al., 2010). For the treatment of ARHL, our previous study has shown that ELZC could restrict cochlear apoptotic damage in mice suffering from ARHL (Dong et al., 2016). However, it is not enough to express ELZC decoction by a single biological process as it is a complex system composed of various chemical compounds.

It is well known that the main character of traditional Chinese medicine is a synergistic effect of various botanical, mineral, or animal drugs to achieve efficiency in the treatment of complex diseases and syndromes. Network pharmacology is an emerging discipline that has been developed in recent years through a combination of pharmacology and bioinformatics based on systems biology, which can maximize the advantages of multi-compound, multi-target, and multi-pathway characteristics of traditional Chinese medicine (Luo et al., 2020). In this study, we applied the network method to construct the network of “herb-active ingredient-action target” (drug-target network) as well as overlap the targets between ELZC and ARHL (drug-disease network). By analyzing the different nodes of these networks, a more comprehensive understanding of ELZC’s effects on age-related hearing loss will be illustrated. Therefore, our study aimed to combine network pharmacology with experiments to explore the potential molecular mechanisms underlying ELZC with a systematic viewpoint, which might shed light upon a novel therapeutic strategy for the prevention and treatment of ARHL.

## Materials and Methods

### Screening Ingredients of ErLong ZuoCi

All the known ingredients of ELZC were identified from the traditional Chinese medicine systems pharmacology database, TCMSP (http://tcmspw.com/tcmsp.php), and related works of literature. The ADME (absorption, distribution, metabolism, and elimination) properties used in this study include oral bioavailability (OB) and drug similarity (DL). The screening conditions were OB greater than 30% and DL greater than 0.18 (Ru et al., 2014).

### Prediction of Targets of the Active Ingredients in ErLong ZuoCi and the Targets Associated with Age-Related Hearing Loss

To predict the putative targets of the pharmacologically active compounds in ELZC, the simplified molecular input-line entry system (SMILES) structures of these compounds were found in the PubChem network database (https://pubchem.ncbi.nlm.nih.gov/) and imported into the Swiss Target Prediction network database (http://www.swisstargetprediction.ch/), which can predict the targets of bioactive molecules (drug-target) based on a combination of 2D and 3D similarity measures with known ligands (Gfeller et al., 2014; Daina et al., 2019). Known ARHL-related targets (disease-target) were obtained from two existing resources: (1) GeneCards database (http://www.genecards.org/); (2) Online Mendelian Inheritance in Man (OMIM) (http://omim.org/) with the keyword “Age-related hearing loss” or “Presbycusis.” The Venn R package was employed to map the intersection genes (drug-disease target) between drug-target and disease-target (Jia et al., 2021).

### Construction and Cluster Analysis of Protein-Protein Interaction Network

PPI of drug-disease targets was derived from String (http://string-db.org/ver.11) (Szklarczyk et al., 2019), an online database of known and predicted protein-protein interactions which were compiled with the criteria of “Homo sapiens,” high confidence (score > 0.7) was set to ensure the reliability of data. Cluster analysis is a classification method that involves interconnected regions showing the inherent pattern in the network. The connected regions in large protein-protein interaction networks may represent molecular complexes (Bader and Hogue, 2003; Barabasi et al., 2011). In this study, we use Molecular Complex Detection (MCODE), a plug-in of Cytoscape v3.6.0 software, to detect densely connected regions and cluster analysis in the PPI network. The criteria settings were set as follows: node score cutoff = 0.2; K-core = 2; and degree of cutoff = 2. Modules with an MCODE score ≥4 and nodes ≥6 were considered for further analysis.

### Visualization and Topological Analysis for Network

The drug-target network and PPI of drug-disease target were constructed by Cytoscape v3.6.0 software, where the nodes independent of the network were excluded. To find the major nodes, topological analysis was performed by the network analyzer module of Cytoscape software. Three topological parameters, “degree” (the number of links to node), “Betweenness Centrality” (the number of shortest paths between pairs of nodes), and “Closeness Centrality” (the mean distance from a node to other nodes) were used to estimate the central properties of the nodes in the network, which correspond to important targets of ELZC that contribute to ARHL.

### Gene Ontology and Kyoto Encyclopedia of Genes and Genomes Pathway Enrichment Analysis

Gene ontology (GO) enrichment analysis including biological process, cell component, and molecular function, as well as Kyoto Encyclopedia of Genes and Genomes (KEGG) pathway enrichment analysis, were performed using the Database for Annotation, Visualization, and Integrated Discovery (DAVID) webserver (https://david.ncifcrf.gov/), the functional enrichment tool. GO terms with *p* value < 0.05 and KEGG pathways with *p* value < 0.05 were considered to have significance.

### Cell Culture

The House Ear Institute-Organ of Corti 1 (HEI-OC1) mouse auditory cell line was obtained from Shanghai Jiao Tong University with approval by Dr. Federico Kalinec (Head and Neck Surgery, David Geffen School of Medicine, University of California, Los Angeles). Cells were cultured in DMEM/high glucose medium with 10% FBS and incubated at 33°C in a 10% CO_2_ incubator.

### Preparation of ErLong ZuoCi

Botanical drugs used to prepare ELZC were purchased from Kangqiao Chinese Medicine Tablet Co., Ltd. The original botanical drugs including Rehmannia glutinosa (Gaertn.) DC. (Orobanchaceae; rehmanniae radix praeparata) (lot# 200307), Cornus officinalis Siebold and Zucc. (Cornaceae; corni fructus) (lot#191231) Dioscorea oppositifolia L. (Dioscoreaceae; dioscoreae rhizoma) (lot#200218), Poria cocos (Schw.) Wolf. (Polyporaceae; Poria) (lot#200121), Alisma plantago-aquatica subsp. orientale (Sam.) Sam. (Alismataceae; alismatis rhizoma) (lot#200120), Paeonia suffruticosa Andrews (Paeoniaceae; moutan cortex) (lot#190810), Bupleurum scorzonerifolium Willd. (Apiaceae; bupleuri radix) (lot#200205), and Magnetitum (lot#190527) were identified by Dr. Hongmei Zhang (School of Pharmacology, Shanghai University of Traditional Chinese Medicine). Drugs used to prepare ELZC decoction were formulated according to the relative proportions as 8:4:4:3:3:3:1:1. According to our previous study (Dong et al., 2016), all crude drugs (total 81 g) were soaked in distilled water (1:8, w/v) for 12 h and decocted to boiling for 1 h. The botanical drug residues were again soaked in distilled water (1:6, w/v) and boiled for 1 h. The two filtrates were finally concentrated to 0.3 g crude drug/mL and filtered through a 0.22 μm size membrane filter known as ELZC extract. The extract was aliquoted and then stored at −20°C until further analysis. For later *in vitro* study, ELZC extract was diluted to the set concentration with phosphate buffer saline before use. To ensure quality control of EZLC, a modified method of high-performance liquid chromatography was applied (Dong et al., 2016). In brief, the quality control of ELZC was completed by the reference standard of Verbascoside, Morroniside, Loganin, 5-hydroxymethylfurfura, Alisol B-23-acetate, Paeonol, which were purchased from the National Institutes for Food and Drug Control (Shanghai, China). The reference compound of Saikosaponin b1 and Saikosaponin b2 were purchased from Nature-Standard Biotechnology (Shanghai, China). These standard compounds were prepared as a control standard for the high-performance liquid chromatography analyses after being precisely weighed and dissolved with methanol. The results are shown in Supplementary Figure S1.

### SA-Beta-Gal Activity Analysis

Auditory HEI-OC1 cells were seeded in a 6-well plate (1.5 × 10^5^ cells/well). At the endpoint of treatment, the medium was removed. In addition, cells were fixed for 15 min with 4% (wt/vol) formaldehyde. Afterward, cells were washed with PBS and stained with senescence-associated *β*-galactosidase staining (SA-*β*-gal) kit (C0602, Beyotime, Biotechnology, Shanghai, China). Photographs were taken using a standard light microscopy (Vert. A1, ZEISS, Oberkochen, German) at 200× magnification. The population of SA-*β*-gal-positive cells was determined by counting 1000 cells in four random fields under microscopy. The results are expressed as the mean of triplicates ±SD.

### Western Blot

Total cell protein was extracted by lysing cells in RIPA lysis buffer with 2% protease and phosphatase inhibitor (Beyotime, China). The protein concentration of each group was determined by bicinchoninic acid (BCA) Protein Assay Kit (P0012, Beyotime Technology, Shanghai, China). A total of 15 μg protein samples were separated by SDS-PAGE and transferred onto PVDF membrane (Merck Millipore, Germany). After blocking with QuickBlock™ solution (Beyotime, China) for 15 min at room temperature, the membranes were incubated overnight (4°C) with specific primary antibodies. The primary antibodies included p21 (SC-6246,1:200, Santa Cruz), p-p53 (#9284, 1:1,000, Cell Signaling Technology), *γ*-H2AX (#9718, 1:1,000, Cell Signaling Technology), AKT (#4691, 1:1,000, Cell Signaling Technology), p-AKT (AF5734, 1:1,000, Beyotime), anti-ERK1/2 (AF1051, 1:1,000, Beyotime), p-ERK1/2 (AF 1891, 1:1,000, Beyotime), anti-Stat3 (AF5315, 1:1,000, Beyotime), anti-p-stat3 (AF1276, 1:1,000, Beyotime). GAPDH (#5174, 1:1,000, Cell Signaling Technology) was used as a loading control. Membranes were then incubated with the appropriate secondary antibody for 1 h at room temperature and visualized by ECL Plus Western Blotting Substrate (NEL105001EA, Waltham, United States) and membranes were imaged by ChemiDOC™ Touch Imaging System (Bio-Rad, Hercules, California, United States) using standard chemiluminescence and analyzed with Image Lab Software (Bio-Rad, Hercules, California, United States). Densitometry was performed by first subtracting the background staining density from each band density. Then, the probing protein/GAPDH ratio was calculated from the band densities in each lane to normalize differences in protein loading. Finally, the ratios of the control and experimental samples were tested for statistical significance.

### Statistics Analysis

Our data are expressed as the means ± standard deviation (SD) and all statistical analyses were performed by GraphPad 7.0 (GraphPad Software, San Diego, CA). One-way analysis of variance (ANOVA) followed by the Dunnett’s post hoc test was used for multiple group comparisons. *p* values <0.05 were considered statistically significant.

## Results

### The Putative Active Ingredients of ErLong ZuoCi

**FIGURE 1:**
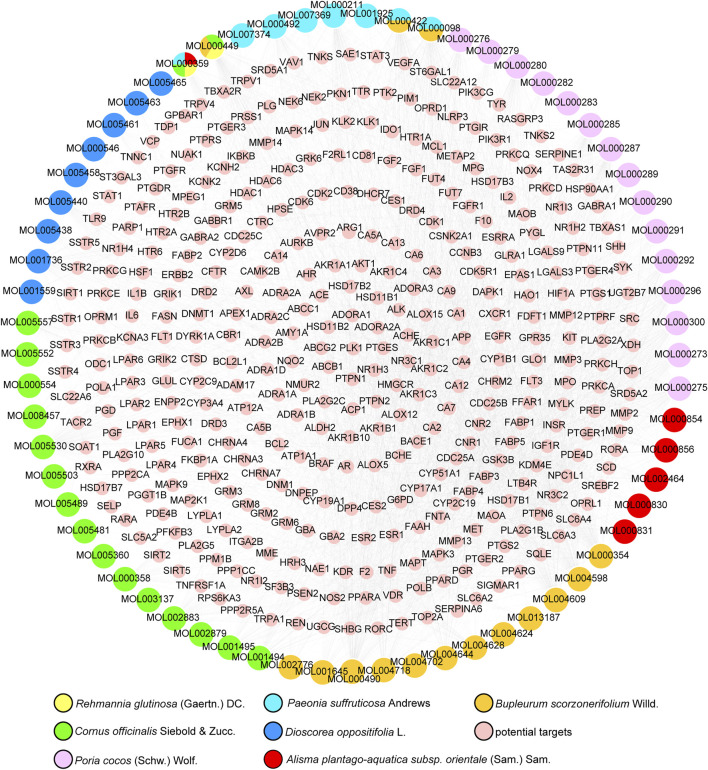
Compound-target network of ELZC. Yellow round nodes represent potential targets of ELZC.

A total of 855 unique compounds were obtained in ELZC, including 76 in Rehmannia glutinosa (Gaertn.) DC (RG), 226 in Cornus officinalis Siebold & Zucc (CO), 71 in Dioscorea oppositifolia L. (DO), 55 in Paeonia suffruticosa Andrews (PS), 34 in Poria cocos (Schw.) Wolf. (PC), 46 in Alisma plantago-aquatica subsp. orientale (Sam.) Sam. (AP), 349 in Bupleurum scorzonerifolium Willd. (BS), 2 in magnet. Oral administration is the main route of TCM drug administration, which is limited by the drug’s ADME characteristics. A total of 73 compounds passed the OB and DL filters and were chosen for further study. Detailed information on active molecules in ELZC is presented in Supplementary Table S1. The SMILES structures of ELZC ingredients obtained from PubChem were imported into the Prediction Target of Swiss Target Prediction network database. Then, a total of 65 chemical compounds yielded 365 unique putative targets, among which 8 candidate compounds had no target. The targets of each compound are listed in Supplementary Table S2. To elucidate the complex interactions of ELZC ingredients and their corresponding targets, we constructed a network based on the potential compounds of ELZC and these compounds’ targets by Cytoscape 3.6.0. The compound-target network consists of 430 nodes (65 compound nodes and 365 compound target nodes) and 2737 edges. The median values for degree centrality (DC), betweenness centrality (BC), and closeness centrality (CC) of all nodes of compounds are respectively 40, 0.357, and 0.018. According to the network analysis results, 22 compounds with DC ≥ median DC, CC ≥ median CC, and BC ≥ median BC were believed to play a key role in this network (Table 1). The compound-target network is shown in Figure 1 and the node interactions of the network and topological analysis are provided in Supplementary Tables S3 and S4. The top 10 compounds with high degree value include quercetin, kaempferol, petunidin, Areapillin, 3′,4′,5′,3,5,6,7-Heptamethoxyflavone, isorhamnetin, Hydroxygenkwanin, Eburicoic acid, trametenolic acid, hederagenin, and dehydroeburicoic acid. We found that 274 of the 365 putative targets were shared between two or more compounds, indicating that these compounds act on some of the same biological processes.

**TABLE 1:** Key compound of ELZC.

No	Mol ID	Molecule name	Betweenness centrality	Closeness centrality	Degree	Herb
C-01	MOL000098	Quercetin	0.049	0.406	102	PS, BS
C-02	MOL000422	Kaempferol	0.056	0.406	102	PS, BS
C-03	MOL000490	Petunidin	0.047	0.401	101	BS
C-04	MOL004609	Areapillin	0.045	0.401	101	BS
C-05	MOL004598	3′,4′,5′,3,5,6,7-Heptamethoxyflavone	0.050	0.401	101	BS
C-06	MOL000354	Isorhamnetin	0.044	0.401	101	BS
C-07	MOL005530	Hydroxygenkwanin	0.060	0.403	101	CO
C-08	MOL000287	3beta-Hydroxy-24-methylene-8-lanostene-21-oic acid	0.046	0.403	92	PC
C-09	MOL000275	Trametenolic acid	0.046	0.403	92	PC
C-10	MOL000296	Hederagenin	0.046	0.394	80	PC
C-11	MOL000300	Dehydroeburicoic acid	0.020	0.389	73	PC
C-12	MOL001736	(-)-Taxifolin	0.093	0.389	73	DO
C-13	MOL002883	Ethyl oleate (NF)	0.023	0.378	67	CO
C-14	MOL004718	Alpha-spinasterol	0.022	0.385	66	BS
C-15	MOL000211	Mairin	0.040	0.388	66	PS
C-16	MOL000292	Poricoic acid C	0.018	0.382	65	PC
C-17	MOL000492	(+)-Catechin	0.060	0.380	63	PS
C-18	MOL000449	Stigmasterol	0.019	0.377	50	RG, CO, BS
C-19	MOL000359	Sitosterol	0.021	0.378	50	RG, CO, AP, PS
C-20	MOL000289	Pachymic acid	0.022	0.366	49	PC
C-21	MOL004644	Sainfuran	0.028	0.369	44	BS
C-22	MOL000276	7,9 (11)-Dehydropachymic acid	0.024	0.365	43	PC

RG = Rehmannia glutinosa (Gaertn.) DC. (RG), CO = Cornus officinalis Siebold and Zucc., DO = Dioscorea oppositifolia L., PS = Paeonia suffruticosa Andrews, PC = Poria cocos (Schw.) Wolf., AP = Alisma plantago-aquatica subsp. orientale (Sam.) Sam., BS = Bupleurum scorzonerifolium Willd.

### Enrichment Analysis Gene Ontology Function and Pathway of Potential Targets of ErLong ZuoCi on Treating ARHL

**FIGURE 2:**
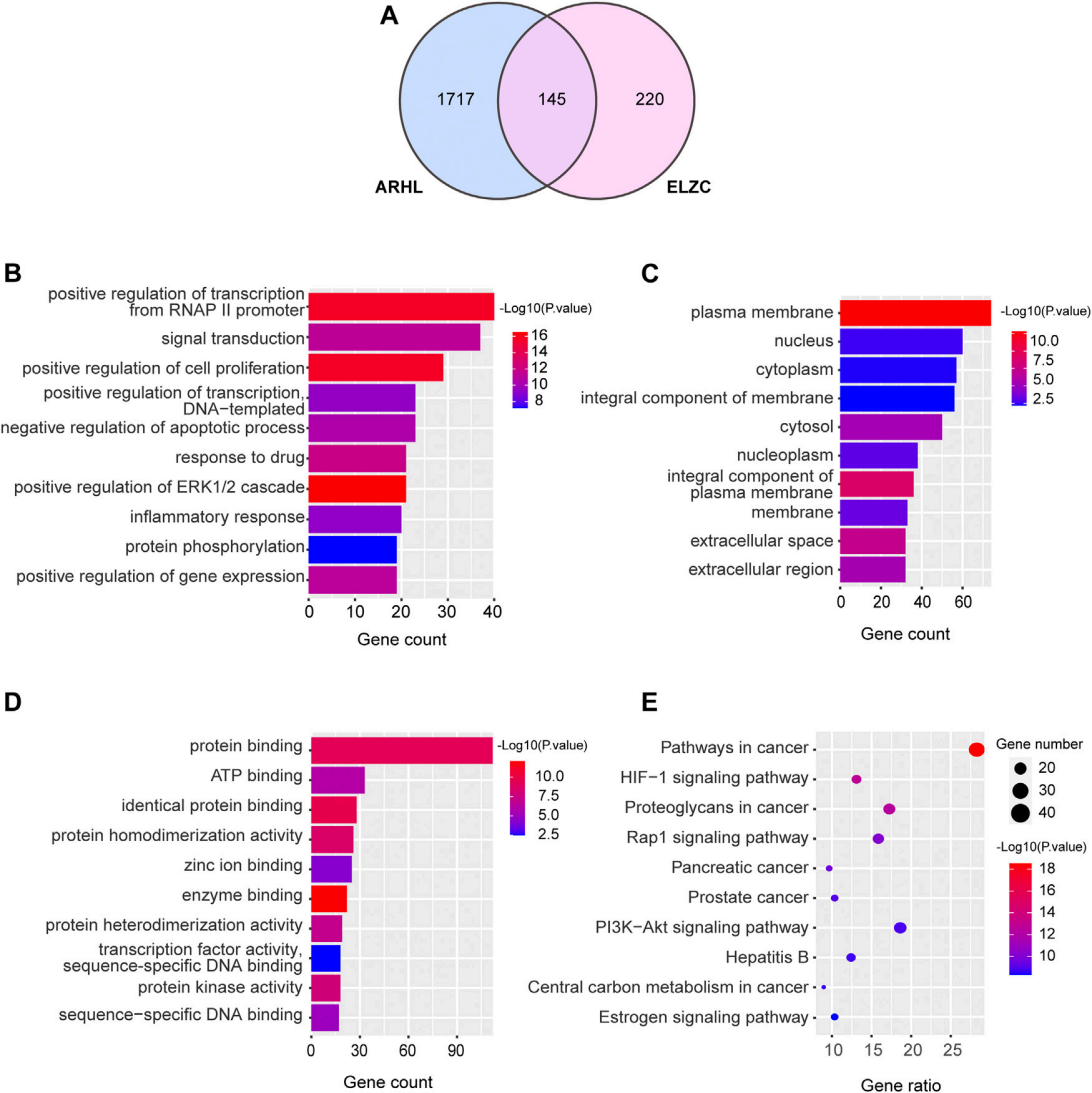
Gene ontology and pathway enrichment analysis of targets in ELZC working on ARHL.The Venn map showed that 145 targets were shared between ELZC and ARHL **(A)**. Enrichment analysis of the biological process **(B)**, cellular composition **(C)**, molecular function **(D)**, and KEGG pathway **(E)** for potential targets of ELZC acting on ARHL were acquired from DAVID functional annotation tool *p* < 0.05.

ELZC had 365 targets obtained from the Swiss Target Prediction network database, and ARHL had 1860 targets collected from the OMIM and GeneCards. Of these targets 145 were shared between ELZC and ARHL (Figure 2A). Namely, these overlapping genes were considered as potential targets of ELZC for treating ARHL. The DAVID database was used to perform GO annotation and KEGG pathway enrichment analysis for potential targets of ELZC action on ARHL. GO annotation enriched in three aspects of molecular function, cell component, and biological process. There were 95 enrichment categories related to molecular function including protein binding and ATP binding; 372 enrichment results that were related to biological process, mainly involved signal transduction, positive regulation cell proliferation, negative regulation of the apoptotic process, regulation of ERK1/2 cascade, immune response, and protein phosphorylation. ELZC’s potential targets were mainly enriched on the plasma membrane, cytosol, and other cell components. Each *p*-value of enrichment results was calculated (*p* values <0.05 were significantly enriched). The top 10 enrichment results are displayed in Figures 2B–D. KEGG pathway analysis showed that the potential targets of ELZC action on ARHL were involved in 102 signaling pathways (*p* < 0.05). These pathways predominantly involved in HIF-1, Rap1, PI3K-AKT, Estrogen, Ras, TNF signaling pathway, etc. Detailed information about enrichment analysis is provided in Supplementary Tables S5–S8. The top 10 KEGG enrichment pathway ranking *p* values according to the order from small to large are displayed in Figure 2E.

### Hub Nodes Screening and Protein-Protein Interaction Network Graph Construction of ErLong ZuoCi Action on Age-Related Hearing Loss

**FIGURE 3:**
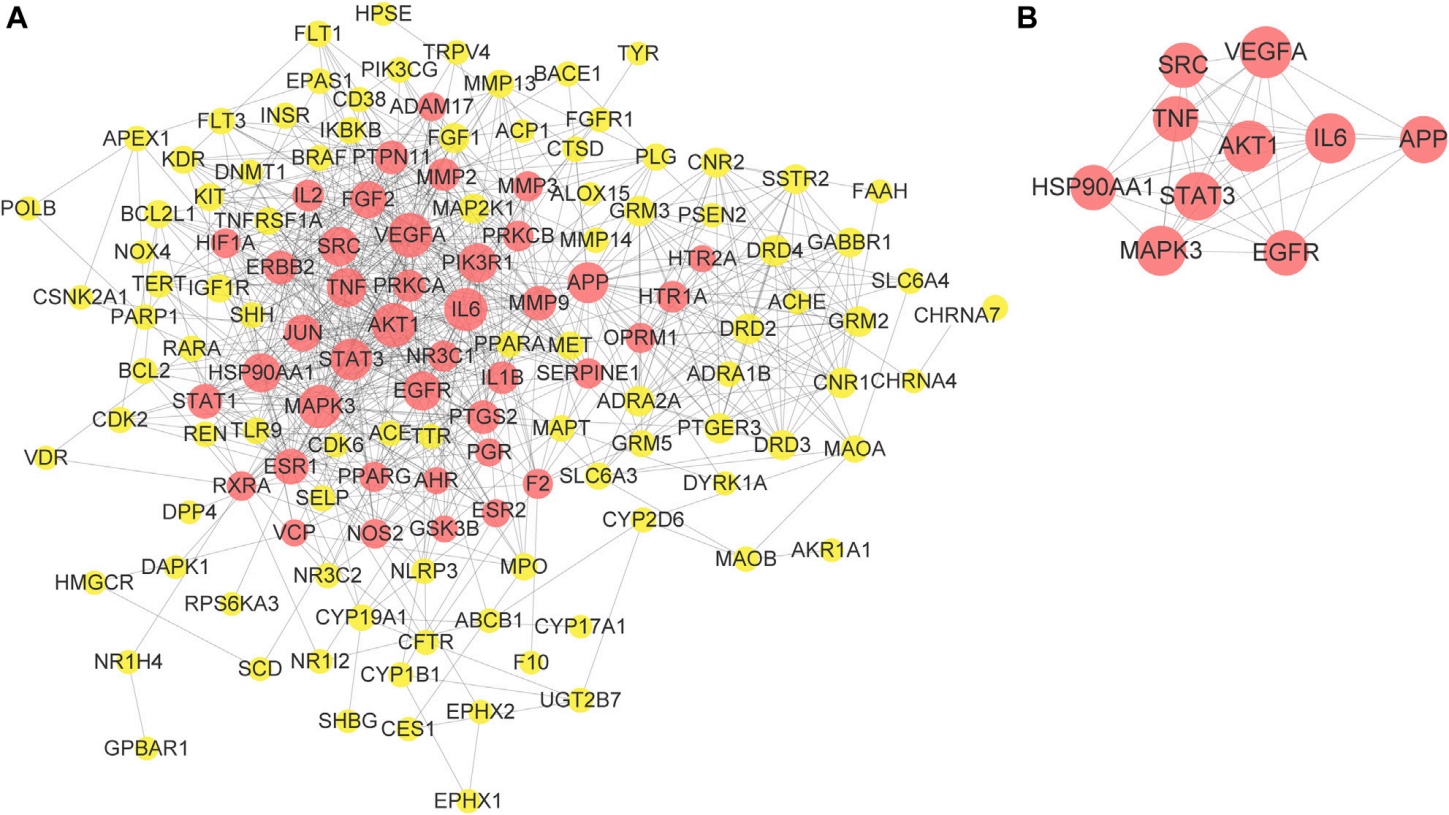
PPI network construction of ELZC action on ARHL. 135-node and 716-edge PPI network of potential targets of the ELZC working on ARHL was acquired at the String database **(A)**. The relationship between the top 10 key targets is visualized by cytoscape software **(B)**. The size of nodes was set according to the degree value. The nodes in red represent key target. The yellow nodes represent the other targets.

To better understand the relationship between the potential targets of EZLC and ARHL, 145 shared targets of ELZC and ARHL were acquired by Venn diagram analysis. The PPI network analysis of the share targets was performed via STRING. A total of 716 interrelations, as well as 135 related targets, are obtained in the PPI network (Figure 3A). Based on a median value of degree ≥8, betweenness ≥0.0036, and closeness ≥0.3851, 41 targets were screened which indicated that ELZC likely influences ARHL via these key targets. Among these hub nodes, VEGFA, AKT1, IL-6, MAPK3, STAT3, APP, TNF, HSP90AA1, EGFR, and SRC are the top 10 targets as listed in Table 2. The relationship between the top 10 key targets is visualized by cytoscape software as shown in (Figure 3B). The topological parameters for all shared targets of ELZC and ARHL are provided in Supplementary Tables S9 and S10.

**TABLE 2:** Top 10 Key targets of ELZC acting on ARHL.

Number	Target name	Betweenness centrality	Closeness centrality	Degree	Protein name
**T-01**	VEGFA	0.088	0.530	43	Vascular endothelial growth factor A
**T-02**	AKT1	0.090	0.504	42	RAC-α serine/threonine-protein kinase
**T-03**	IL6	0.071	0.525	40	Interleukin-6
**T-04**	MAPK3	0.095	0.536	40	Mitogen-activated protein kinase 3
**T-05**	STAT3	0.039	0.506	37	Signal transducer and activator of transcription 3
**T-06**	APP	0.198	0.500	35	Amyloid-beta precursor protein
**T-07**	TNF	0.047	0.502	32	Tumor necrosis factor
**T-08**	HSP90AA1	0.047	0.482	32	Heat shock protein HSP 90-α
**T-09**	EGFR	0.056	0.513	32	Epidermal growth factor receptor
**T-10**	SRC	0.049	0.479	32	Proto-oncogene tyrosine-protein kinase Src

### Molecular Complex Detection Cluster Analysis

**FIGURE 4:**
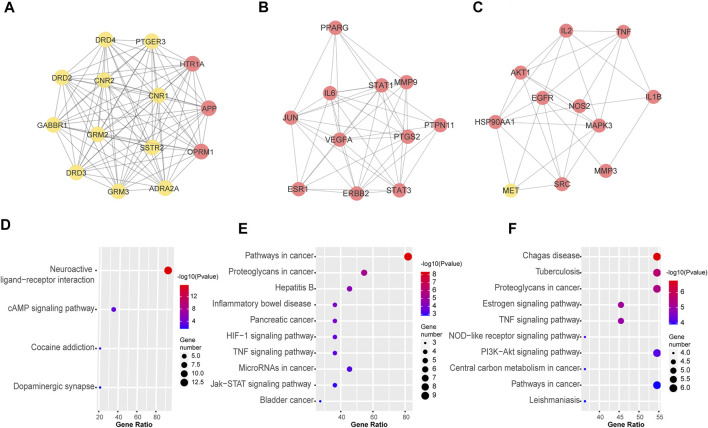
Top three sub-networks with high scores from the network of PPI by MOCDE cluster analysis. MCODE algorithm was applied to identify the densely connected network components. Three powerful functional networks were identified. **(A)** The subnetworks 1 includes 14 nodes and 91 edges; **(B)** The subnetwork 2 includes 11 nodes and 45 edges; **(C)** The subnetwork 3 includes 11 nodes and 30 edges. **(D-F)** Representative terms of KEGG pathway for nodes from subnetworks 1, 2, and 3. The nodes in red represent important targets of the ELZC working on ARHL.

Next, we used MCODE to screen the top three sub-networks with high scores from the network of PPI. A total of three tightly connected network clusters were identified with MCODE score ≥4 and nodes ≥6. Module 1 contained 14 nodes and 91 edges (Figure 4A). By analyzing the relevant biological processes, molecular functions, and cellular components of every cluster, we found that cluster 1, which was enriched in the tissue of the brain and brain cortex, mainly concentrated on the biological process of chemical synaptic transmission; molecular function on the dopamine neurotransmitter, glutamate receptor activity, and channel regulator activity for calcium and potassium. The KEGG enrichment analysis provides support for the pharmacological effects of cluster 1 being associated with neuroactive ligand-receptor interaction and cAMP signaling pathway (Figure 4D). The other two clusters, Module 2 with 11 nodes and 45 edges (Figure 4B) and Module 3 containing 11 nodes and 30 edges (Figure 4C) were enriched in HIF-1, JAK-STAT and TNF signaling pathway, PI3K-Akt, Estrogen, NOD-like receptor signaling pathway, and other pathways, as shown in Figures 4E,F. Details of each cluster enrichment analysis are described in Supplementary Tables S11 and S12.

### Experimental Validation of ErLong ZuoCi Decreasing Auditory Cell Senescence

**FIGURE 5:**
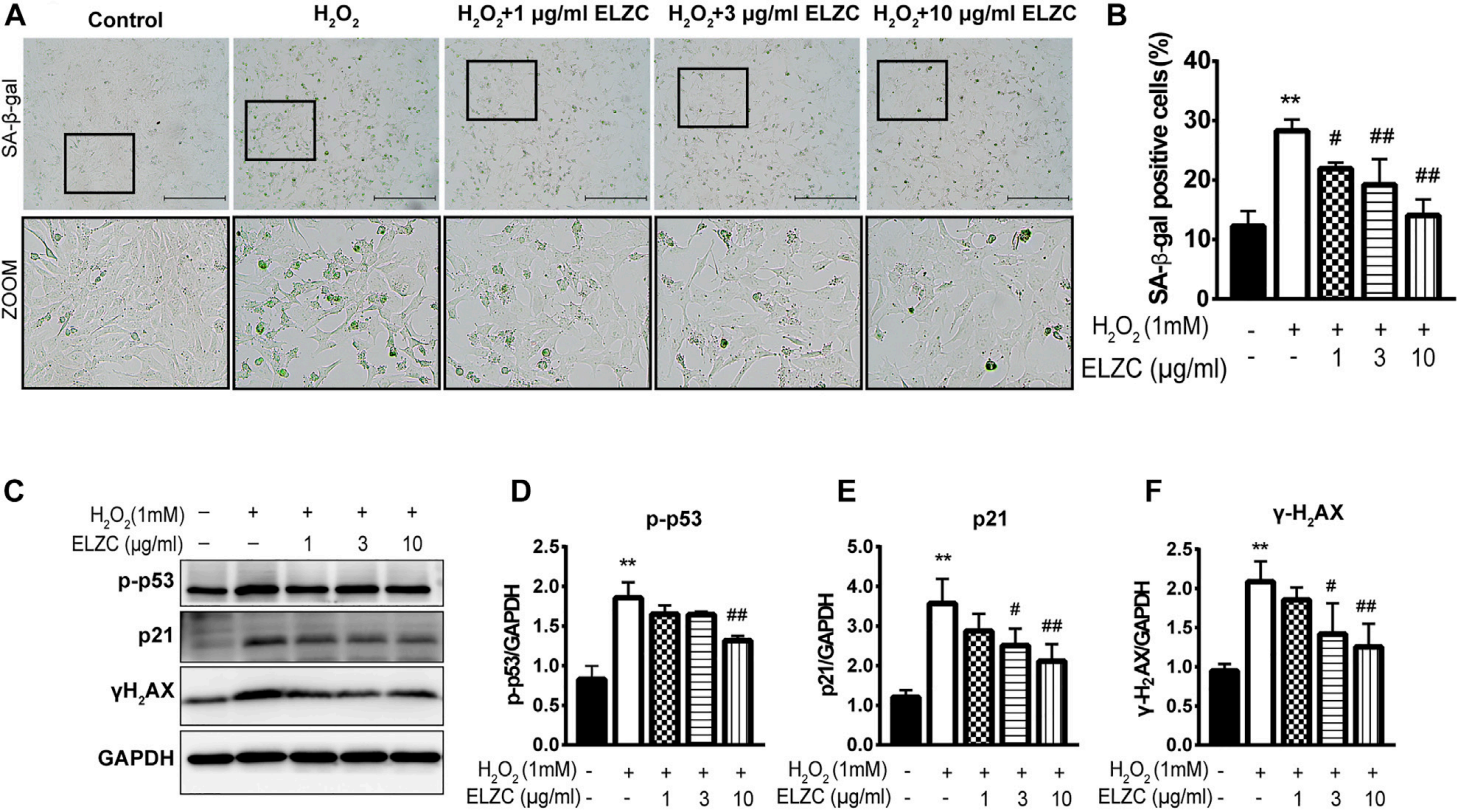
ELZC prevents from H_2_O_2_-induced cellular senescence in auditory hair cells. **(A)** Hei-oc1 cells were exposed to H_2_O_2_, and SA-β-gal activity stain assay representative image of H_2_O_2_-induced Hei-oc1 cells pretreated with a serial concentration of EZLC for 24 h are shown. Scale bar represents 200 μm; **(B)** number of SA-*β*-gal positive cells (*n* = 5). The values represent the mean ± SD. *p < 0.05 and **p < 0.01 vs. control; ##*p* < 0.01 vs. H_2_O_2_ (analysis of variance using Dunnett’s post-hoc test); **(C–F)** Western blotting of cellular senescence-related protein, p21, p-p53, γH2AX in H_2_O_2_-exposed hei-oc1 cell treated with ELZC (*n* = 3). The values represent the means ± SD; **p < 0.01 vs. control, #*p* < 0.05 and ##*p* < 0.01 vs. H_2_O_2_ (analysis of variance, using Dunnett’s post-hoc test).

To examine the protective effect of EZLC on ARHL, we set up H_2_O_2_ induced-senescence phenotype cell model (Tsuchihashi et al., 2015; Kamogashira et al., 2017) to resemble as closely as possible the *in vivo* environment of ARHL. The Hei-oc1 cells were pretreated with 1, 3, and 10 μg/ml ELZC for 24 h. Then the cells were exposed to H_2_O_2_ (1 mM) for 1 h. Our results revealed that cellular senescence was induced 24 h after H_2_O_2_ treatment at a rate of 28.27 ± 1.94% HEI-OC1 cells stained with *β*-gal staining, which is widely used as the cell senescence-associated marker (colored in blue-green in Figure 5A and the quantification analysis is shown in Figure 5B) (Fuhrmann-Stroissnigg et al., 2019). Only 12.28 ± 2.51% of SA-*β*-gal-positive Hei-oc1 cells were seen in the control group. In ELZC-pretreated cells, the SA-*β*-gal-positive cell rate was significantly reduced to 14.07 ± 2.71%. Next, the expression levels of p16^INK4a/Rb^, p21, p-p53, which correlates with the induction of cellular senescence markers (Kuilman et al., 2010) were detected by Western blot analysis. H_2_O_2_ significantly upregulated p21, p-p53 expression, which was blocked by pretreatment of ELZC (Figures 5C–E). Meanwhile, the level DNA damage marker γ-H2AX (Mah et al., 2010), an important trigger in the aging process, was also decreased (Figures 5C–F) in ELZC-pretreated cells. These results reveal that ELZC could either delay or reverse the population of senescent cells.

### ErLong ZuoCi Upregulates Extracellular Signal-Regulated Kinase (ERK) and Downregulates STAT3 Expression in Auditory Hair Cells

**FIGURE 6:**
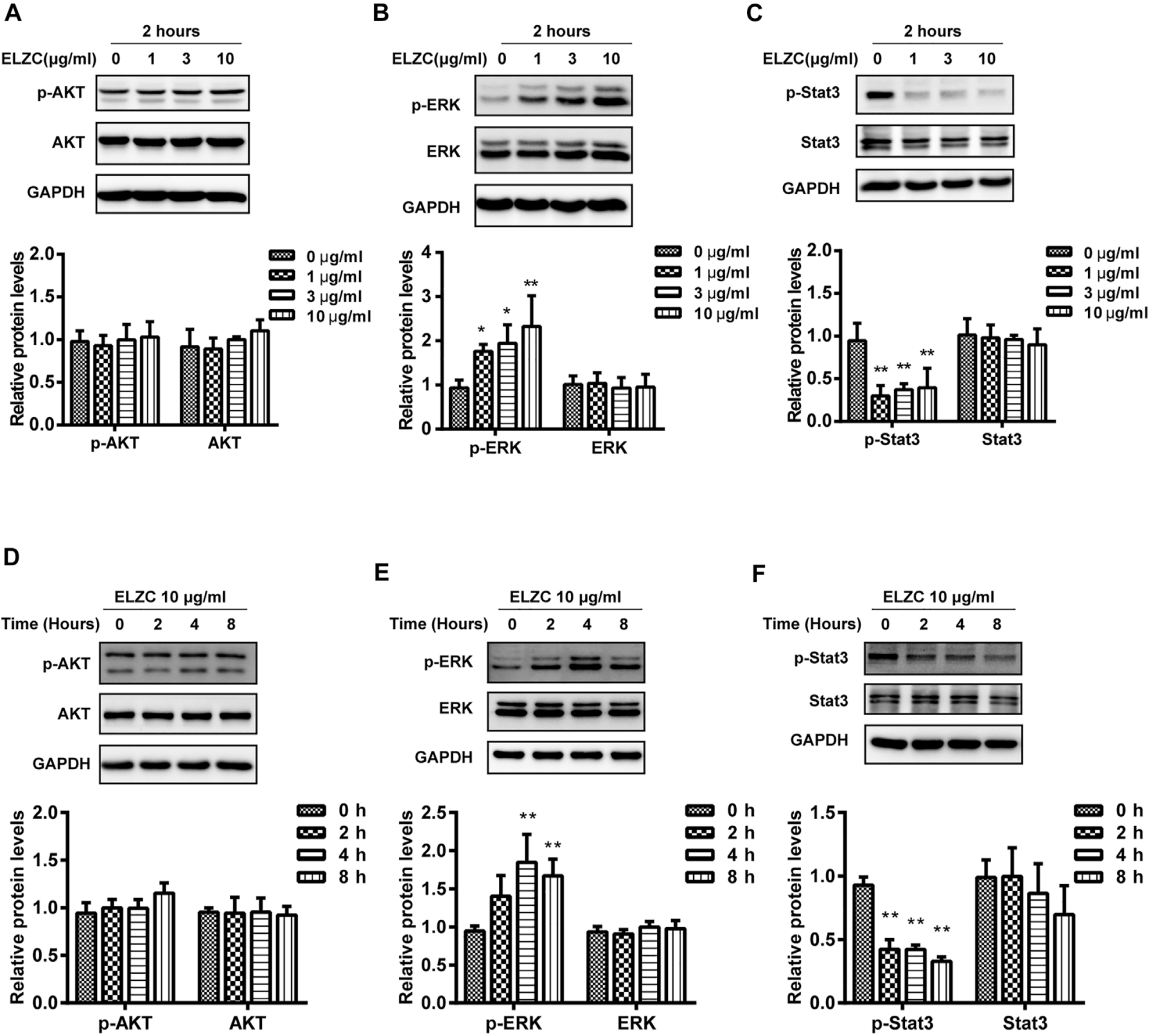
Experiment validation key targets of ELZC working on age-related hearing loss. Hei-oc1 cells were treated with ELZC (1, 3, 10 μg/ml) for 2 h **(A–C)**. In another round of experiments, Hei-oc1cells were treated with 10 μg/ml ELZC for 2, 4, 8 h **(D–F)**. Protein expression of key targets, including AKT, p-AKT, ERK, p-ERK, STAT3, and p-STAT3 was detected by Western blot (*n* = 3). Expression of GAPDH serves as a loading control and band intensities were quantified by Image Lab 6.0 followed by the analysis of variance, using Dunnett’s post-hoc test at *p*<0.05.

To verify the predicted targets of ELZC for the treatment of ARHL, we evaluated the expression levels of AKT, ERK1/2, STAT3, and their phosphorylation level by Western blot. A total of 10 μg/ml ELZC slightly increased the p-AKT at Thr308 (Figure 6A) but not at Thr473 (data not shown) compared to the control group. As shown in Figure 6B, the expression of p-ERK was increased and p-STAT3 was reduced significantly in Hei-oc1 cells treated with 1–10 μg/ml ELZC for 2 h, while the expression of total ERK1/2 or STAT3 was not altered (Figures 6B,C). Then, Hei-oc1 cells were incubated with 10 μg/ml of ELZC for 0, 2, 4, and 8 h, respectively. After 2–8 h incubation of ELZC, there’s no significant change in the expression of p-AKT when compared to control group (Figure 6D). ELZC increased the expression of p-ERK and sustained its expression until around 4 h (Figure 6E). On the other hand, treatment with ELZC resulted in rapid and sustained attenuation of p-STAT3 (Figure 6F).

### Involvement of ERK in ErLong ZuoCi’s Protection Effect in Hydrogen Peroxide-Induced Auditory Hair Cell Model

**FIGURE 7:**
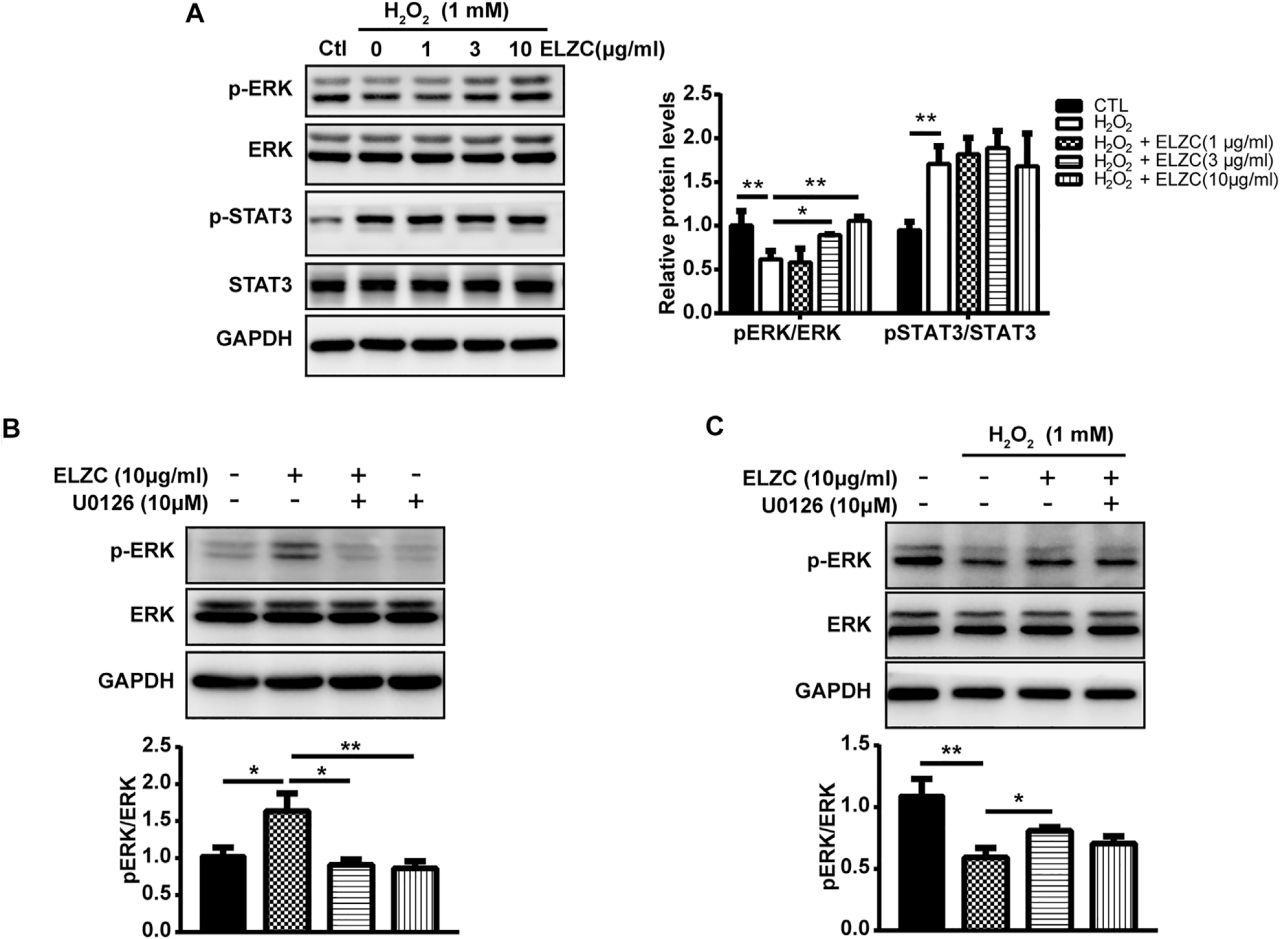
Involvement of ERK in EZLC’s protection effect in hydrogen peroxide-induced auditory hair cell model. The Hei-oc1 cells were pretreated with 1, 3, 10 μg/ml ELZC for 24 h. Then the cells were exposed to H_2_O_2_ (1 mM) for 1 h. Protein expression of key targets, including ERK, p-ERK, STAT3, and p-STAT3 was detected by Western blot **(A)**; Hei-oc1cell were concomitant treatment with ELZC and U0126 (10 μM) in normal culture medium **(B)** or under oxidative stress induced by H_2_O_2_ treatment. **(C)** Protein expression of ERK, p-ERK was detected by Western blot. Expression of GAPDH serves as a loading control and band intensities were quantified by Image Lab 6.0 followed by the analysis of variance, using Dunnett’s post-hoc test (*, *p* < 0.05; **, *p* < 0.01).

To further verify the predicted targets in H_2_O_2_ induced auditory hair cell model, the activation of ERK and inhibition of STAT3 were monitored by Western blot analysis using p-ERK/ERK and p-STAT3/STAT3. The Hei-oc1 cells were pretreated with 1, 3, and 10 μg/ml ELZC for 24 h. Then the cells were exposed to H_2_O_2_ (1 mM) for 1 h. H_2_O_2_ decreased the expression of active ERK1/2 (phosphor-ERK1/2), on the contrary, ELZC pretreatment promoted the phosphorylation of ERK1/2 (Figure 7A). For the expression of p-STAT3, H_2_O_2_ significantly increased its expression. But ELZC pretreatment failed to inhibit the expression of p-STAT3 induced by the H_2_O_2_ treatment. To explore the contribution of ERK in ELZC’s protecting effect on auditory hair cell, we applied a specific p-ERK inhibitor U0126 to challenge the Hei-OC1 cell that is with or without H_2_O_2_ treatment. As shown above, ELZC induces p-ERK expression in Hei-OC1 cells, while concomitant treatment with U0126 ameliorates the enhancement of p-ERK by ELZC, which suggests an important role for ERK (Figure 7B). In a further round of experiments, we determined whether U0126 could similarly block p-ERK with the presence of H_2_O_2._ Although U0126 showed a trend to reduce enhancement of p-ERK by ELZC, we did not see a significant drop in the group of ELZC in combination with U0126, compared to the ELZC group (Figure 7C).

## Discussion

In our network pharmacology where 73 compounds were retrieved after screening ELZC for oral bioavailability and drug-likeness, 22 compounds were predicted to hub nodes in the compound-target network which degree values are greater than the medium by analyzing the topological characters. Some chemical compounds have been reported for their protective effects on the auditory system (Hirose et al., 2016; Akefe et al., 2020). For example, Quercetin is found in two botanical drugs of ELZC that are Paeonia suffruticosa Andrews and Bupleurum scorzonerifolium Willd. Many studies have reported that Quercetin reduced the symptoms of age-related diseases, such as osteoarthritis and age-related macular degeneration (Shao et al., 2019) by upregulating antioxidant enzymes, reducing the level of reactive oxygen species (ROS), and reversing mitochondrial dysfunction. Studies regarding the beneficial effect of quercetin on the inner ear have been reported by several research groups as well. Quercetin can significantly protect hair cells survival against ROS-mediated cisplatin (Lee et al., 2015) or gentamicin (Sagit et al., 2015) -induced damage in a zebrafish lateral line and rat cochlear. Yoshinobu et al.(Hirose et al., 2016) found administrating Quercetin (100 mg/kg/day) to rodents for 3 days prior to and 3 days after noise exposure can improve auditory function of guinea pig with the evidence that the auditory threshold shift is reduced around 15 dB. Furthermore, Lewinska et al. (2020) reported quercetin can specifically decrease the number of senescent cells and suppress the senescence-associated pro-inflammatory response, indicating that quercetin may be considered as a promising candidate for age-related disease therapies. Kaempferol has been investigated for multiple pharmacological effects in antioxidant (Choi, 2011) and anti-inflammatory (Chen et al., 2012; Wang et al., 2020). As summarized in a recent review (Silva Dos Santos et al., 2020), kaempferol may possess beneficial effects in neurodegenerative diseases such as Alzheimer’s and Parkinson’s disease. Akefe et al. (2020) recently demonstrated that a single treatment of kaempferol or in combination with Zn improved neurobehavioral performance, increased the activity of brain catalase, GPx, and SOD, in Wistar rats that were exposed to a precious sound to 100 dB at frequencies in the range of 0–20 kHz. Other chemical compounds such as Poricoic acid C and dehydroeburicoic acid, the major chemical constituents found in *Poria Cocos (Schw.)* Wolf. Lu et al.(2021) have been reported to involve the modulation of redox signaling including the inhibition of pro-inflammatory nuclear factor kappa B signaling and its target genes as well as the activation of antioxidative nuclear factor-erythroid-2-related factor 2 signaling and its downstream target gene in TGF-β1. Alisol B (acetate), Alisol C (monoacetate) account for 65% of chemical compounds identified from *Alisma Orientale (Sam.)* Juz (Qian et al., 2018) and have been widely investigated for its anti-inflammation effect. The promising anti-inflammatory and anti-oxidant activity from these compounds might contribute to ElZC in the treatment of ARHL.

As demonstrated in Figure 2A, the Venn map revealed 145 intersection targets on ELZC and ARHL, which were considered to be the potential targets of ELZC on treating ARHL. GO function and KEGG pathway enrichment analysis indicated that these intersection targets are involved in signal transduction, positive regulation of cell proliferation, negative regulation of apoptotic process regulation of ERK1/2 cascade, immune response, and protein phosphorylation, clearly close to age-related hearing loss. After analyzing the PPI network of 145 potential targets of ELZC working on ARHL, we found that VEGFA, AKT1, IL-6, MAPK3, STAT3, APP, TNF, HSP90AA1, EGFR, SRC, and so on may represent hub targets against ARHL. MCODE is a classic clustering algorithm for identifying the protein complexes from the given network as a whole, especially used to predict key targets in the protein interaction network (Nakajima et al., 2021). Based on the centrality of the network, the complexes generated from this clustering algorithm will be automatically scored, ranked, and further processed to create a cluster network (Bader and Hogue, 2003). In this study, we applied MCODE cluster analysis and found that ELZC contained a total of three clusters as shown in Figure 4. Module 1 cluster from ELZC contains as many as 14 genes (APP, ADRA2A, CNR1, OPRM1, HTR1A, GABBR1, GRM2, PTGER3, DRD4, DRD3, SSTR2, DRD2, CNR2, and GRM3) mainly regulating synaptic transmission by encoding neurotransmitter receptors, such as dopamine receptor, glutamate receptor, and cannabinoid receptor, in the “Neuroactive ligand-receptor interaction” pathway and cAMP pathway. Furthermore, we found the axon dendrite presynaptic membrane and post-synapse are the main cellular components of cluster 1 which are different from the other two clusters. The model 2 cluster containing 11 targets (PPARG, MMP9, IL6, ESR1, STAT1, ERBB2, STAT3, VEGFA, PTGS2, JUN, and PTPN11) mainly participate in the HIF-1 signaling pathway, TNF signaling pathway, Jak-STAT signaling pathway, all of which are involved in the inflammation process. Model 3 cluster from ELZC containing 11 targets (NOS2, TNF, MET, IL2, MMP3, EGFR, SRC, HSP90AA1, MAPK3, IL1B, and AKT1) participating in the signaling pathway of estrogen, TNF, and PI3K/ATK pathway, which revealed that Model 2 and Model 3 clusters were linked to cell proliferation and inflammatory response. For decades, it was assumed that cochlea neuron loss occurred only after hair cell death, thus hair cells have been the primary damage targets in sensorineural hearing loss via inflammation, apoptosis, cell growth arrest, etc. The auditory nerve loss was rarely of functional significance in ARHL. However, this classic view has been challenged in recent years. As it is known, sound-evoked vibration of the basal membrane in the cochlea will produce the motion of hair cell stereocilia, which open and close transduction channels therein. Synaptic connections between hair cells and cochlear neurons might be destroyed well before the hair cells are damaged (Maison et al., 2012; Liberman, 2017; Du et al., 2020). The interesting new insights into the MCODE analysis is that the GO function by module 1, including DA binding and receptor activity, glutamate receptor activity, calcium channel regulator activity, and potassium channel regulator activity, all support the notion that ELZC may function as a modulator of chemical synaptic transmission in auditory pathways.

For the *in vitro* study, the protective effect of EZLC on ARHL was evaluated in auditory Hei-oc1 cells. Cell senescence is one of the hallmarks of aging known to negatively influence age-related diseases including age-related hearing loss. Current experimental evidence supports that the population of senescent cells that persist in tissues can induce aging and aging-associated diseases (McHugh and Gil, 2018; Tchkonia and Kirkland, 2018). Drugs that selectively kill senescent cells may improve age-related symptoms. We observed that ELZC is able to attenuate SA-*β*-gal activity, one of the best-characterized markers for the detection of senescent cells. Our results demonstrate that ELZC could reduce DNA damage, evidenced by a decreased expression of γ-H2AX in Hei-oc1 cells treated by ELZC. Cellular senescence is originally found as a stable cell cycle arrest response to accumulated oxidative stress and DNA damage. This growth arrest is implemented by the activation of p16^INK4a/Rb^ and/or p53/p21^CIP1^ tumor suppressor networks (Kuilman et al., 2010) We found pre-treatment of ELZC can reduce the level p21 and p53, which revealed that ELZC may attenuate cellular senescence. Consistent with and building upon previous reports, our results show that treatment with ELZC attenuates age-related hearing loss *in vivo* and *in vitro*. We speculate the pharmacological action of ELZC might be related to restricting cell death and attenuate cellular senescence in the auditory hair cells. The underlying mechanism is our subsequent research direction.

IL-6, STAT3, TNF, MAPK3, and AKT1 are ELZC’s key targets as predicted by network pharmacology analysis. They all participate in the pathways related to cell proliferation, cell senescence, and senescence-associated inflammation. ERK1 and ERK2 (ERK1/2) were the first MAPKs to be identified and are encoded by two genes MAPK3 and MAPK1, respectively. The phosphorylation of ERK1/2 results in the activation of its kinase activity and leads to pro-survival factors (Xia et al., 1995; Rai et al., 2019). Recently, Battaglia et al. (2003) found that ERK1 and ERK2 activation by long-term blockade of ERK’s specificity protein kinases (MEK1/2), can cause auditory hair cell death. From this perspective, the activation of ERK1/2 in hair cells is considerable for their survival. Further study by Kurioka (Kurioka et al., 2015) confirmed the role of ERK2 in the cochlea that hair cell-specific ERK2 conditional knockout mice have higher auditory thresholds, manifesting in worse hearing loss compared to control mice after noise exposure. Recent studies also showed that Metformin can attenuate hearing loss and rescue age-related neurodegeneration by up-regulation of ERK1/2 signaling pathway in aging mice (Cai et al., 2020). These results all support that ERK1 and 2 are of vital importance in hearing protection. Consistent with our functional analysis of the biological processes, high expression of phosphorylated EKR in the ELZC group indicated the activation of the ERK1/2 signal transduction pathway. Therefore, it is worth emphasizing that ELZC, which is mediated by the ERK1/2, is crucial in preventing degeneration of auditory hair. Chronic inflammation is now understood to be a common characteristic of aging tissues. The non-sensory area of the cochlear (the stria vascularis and the spiral ligament areas) and sensory hair cells will acquire an increased inflammatory state with aging (Watson et al., 2017). In the present study, network pharmacology allowed us to predict that ELZCs would be involved in the pathway of modulating the HIF-1 signaling, TNF signaling, and Jak-STAT linking to immune response. Among those key targets, activated STAT3 plays a critical role in the production of various pro-inflammatory cytokines such as IFN-γ, TNF-α, and NF-κB (Satoh et al., 2003; Landegger et al., 2019; Fetoni et al., 2021), can infiltrate cells of the inner ear and induce damage to the cochlea. As a result, in auditory hair cells, we selected the three key nodes of AKT, ERK1/2, and STAT3 as potential targets for the next experimental verification.

Then, we investigated whether ELZC can activate ERK1/2 and be verified by ERK1/2 inhibitor U0126. Our data revealed that ELZC can activate ERK1/2 phosphorylation in Hei-oc1 cells which were incubated with (Figure 7A) or without 1 mM H_2_O_2_ (Figures 6B,F). Of note, ERK inhibitor significantly attenuated the ELZC-induced p-ERK expression in Hei-oc1 cells that were treated with normal culture medium (Figure 7B), in contrast, a less antagonistic effect was observed in cells treated with H_2_O_2_ (Figure 7C). The main possible explanation for this heterogeneity is that extracellular sources generating an uncontrolled increase of ROS can modify multiple proteins including protein phosphorylation and avoid protein recycling. Therefore, ELZC contributes less on ERK phosphorylation after H_2_O_2_ treatment compared with its contribution without H_2_O_2_ treatment. What is more, since the overproduction of ROS can promote incorrect proteins modification, disruption of mitochondrial integrity leading to apoptosis and possible necrosis, it is difficult to rescue auditory hair cells from oxidative stress damage. Thus, the data present here addresses the importance of intervention on Hei-oc1 hair cells before ROS damage. As it is known that active ERK1/2 is highly effective in enhancing antioxidant defense system, comprised of antioxidant enzymes such as reduced glutathione, superoxide dismutase, and catalase. These enzymes are capable of neutralizing ROS that are generated from normal hair cell activity or oxidative stress condition (Li and Holbrook, 2003; Zhou et al., 2018). So that we deem the pretreatment of ELZC might strengthen antioxidant enzyme activity which can be ascribed to ERK1/2 phosphorylation. Further research along these lines is needed. Our pharmacological experiment data showed that ELZC could significantly decrease the level of p-STAT3 in the Hei-oc1 cells in a dose-dependent and time-dependent manner as shown in Figures 6C,F, which means STAT3 might be a key target for ELZC. However, no similar reduction effect was observed in the H_2_O_2_-induced Hei-oc1 cells (Figure 7A). Previous studies on the senescent processes which contribute to ARHL have focused primarily on degenerative changes in H_2_O_2_-stimulated cochlear hair cells (Qi et al., 2019; Zhang et al., 2021). In contrast to the cochlear hair cell Hei-oc1, the lateral wall is known for “vascular rich” environment and stria vascularis is believed to block immune cells, inner ear antigens, and antibodies. Thus, researchers assumed that the lateral wall and stria vascularis might be more responsible for immune-mediated hearing loss (Fujioka et al., 2014). Such hypothesis recently has been confirmed by immunohistochemistry analysis of age-graded series of the human cochlear. Results demonstrated that the infiltrating immune cells-macrophages in the auditory nerve and cochlear lateral wall showed the population and morphological change according to human aging (Noble et al., 2019). Therefore, whether the depression of STAT3 by ELZC could help to prevent senescence-associated inflammatory and whether it is achievable through the lateral wall or stria vascularis needs to be further explored *in vivo*.

**FIGURE 8:**
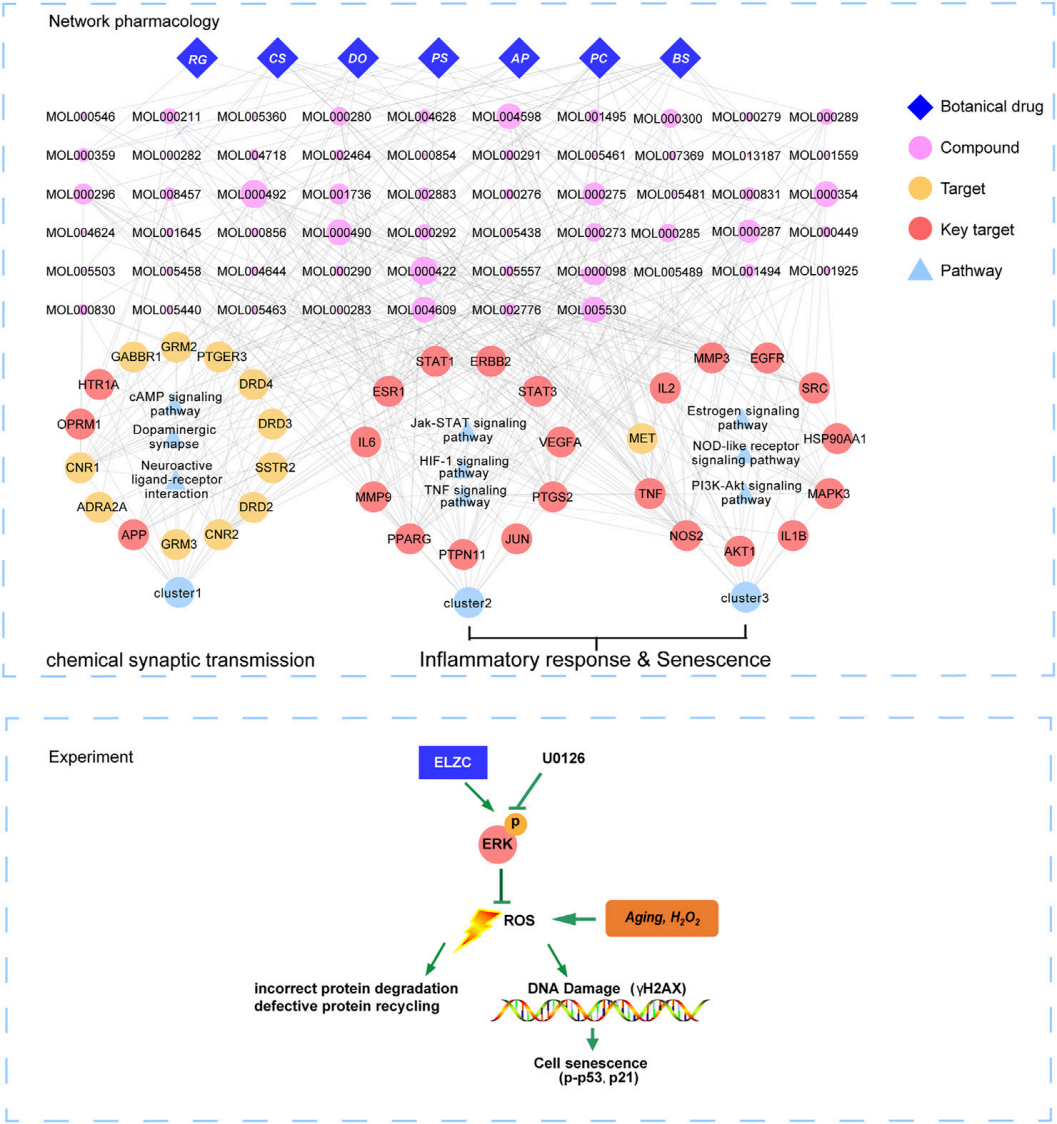
The possible targets/pathways underlying protective effect of ELZC for ARHL. Blue diamond nodes represent each herb of the ELZC. The pink nodes represent candidate active compound and node size is proportional to their degree. The yellow nodes represent target of ELZC acting on ARHL, the red nodes represent key targets. Blue triangle nodes represent the main pathway enrichment. Edges represent interaction among botanical drugs, target, signaling pathway, and functional modules.

The multiple components of TCM formulas remain the major obstacle to explain the mechanism of action of these drugs on various diseases. Network pharmacology provides a multi-dimensional perspective to understand the mechanism of formulations. According to this approach, we found that targets of ELZC contribute to the amelioration of ARHL via three elements of the cochlear aging process: increasing synaptic connections, regulating inflammation and attenuating degeneration of the auditory hair cells (Figure 8). Further experiment data illustrate that ELZC’s prevention on the degeneration of cochlear may be linked to ERK activation. However, there are limitations for the use of network pharmacology to predict active ingredients and potential mechanisms of action of certain drugs. First, the active ingredients screened might not be bioavailable in ARHL patients taking ELZC. Second, it is difficult to distinguish inhibitory effects from activated effects of the targets, and the different dosages of compound might have different impacts on the target as well; Furthermore, target identification usually relies on a database that may be biased due to highly studied pathways/functions. To enhance the reliability of the results, further studies are needed to validate the potential effective target/pathway for ARHL amelioration.

## Data Availability

The original contributions presented in the study are included in the article/Supplementary Material. Further inquiries can be directed to the corresponding author.
