# Supplementary figures and images for: Prediction of the Molecular Mechanisms Underlying Erlong Zuoci Treatment of Age-Related Hearing Loss via Network Pharmacology-Based Analyses Combined with Experimental Validation
Source: Front Pharmacol. 2021 Nov 23;12:719267. doi: 10.3389/fphar.2021.719267 (PMC8650627; doi:10.3389/fphar.2021.719267)

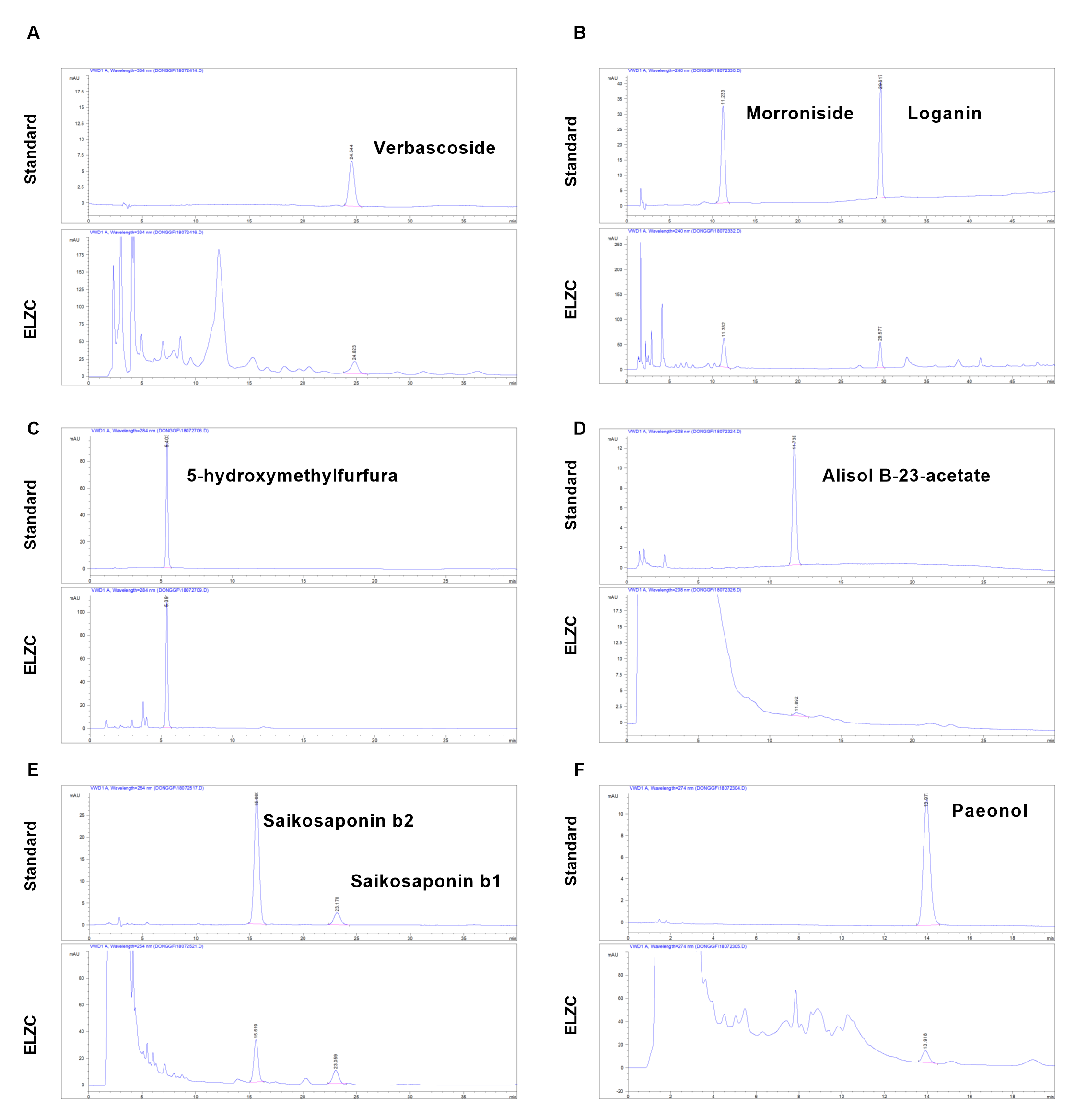

Supplement: Supplementary file 3 [file Image1.TIF]
